# Anxiety and Depression in Breast Cancer Patients Before and After Chemotherapy: A Pre–Post Study Without a Control Group

**DOI:** 10.3390/jcm14228105

**Published:** 2025-11-16

**Authors:** Magdalena Konieczny, Jolanta Sawicka, Izabela Gąska, Elżbieta Kaczmar, Małgorzata Pasek, Agnieszka Kiedik, Łukasz Rypicz, Dorota Kiedik

**Affiliations:** 1 Medical Institute, Jan Grodek State University in Sanok, 38-500 Sanok, Poland; 2 Department of Nursing, Faculty of Health, University of Applied Sciences in Tarnów, 33-100 Tarnów, Poland; 3 Faculty of Medicine, Medical University of Gdansk, 80-210 Gdansk, Poland; 4 Department of Public Health, Division of Public Health, Faculty of Health Sciences, Wroclaw Medical University, 50-367 Wroclaw, Poland

**Keywords:** anxiety, depression, breast cancer

## Abstract

**Background:** Emotional disturbances such as anxiety and depression are common in breast cancer patients and may intensify during systemic therapy. This study aimed to assess changes in the severity of anxiety and depression among women undergoing neoadjuvant chemotherapy and to identify factors influencing emotional outcomes. **Methods:** A total of 211 women with stage I–III breast cancer treated at the Podkarpackie Oncology Center in Brzozów, Poland, were included. Anxiety and depression were assessed using the Hospital Anxiety and Depression Scale (HADS) one week before and three weeks after chemotherapy. Statistical analyses were performed using the Wilcoxon signed-rank test and descriptive statistics (STATISTICA v.13). **Results:** After chemotherapy, anxiety and depression levels increased significantly. Nearly half of the patients experienced clinically relevant anxiety, and over one-third showed symptoms of depression. The emotional burden appeared particularly high among women reporting financial difficulties. In contrast, no clear associations were found with marital status, place of residence, or cancer stage. **Conclusions:** Chemotherapy in breast cancer patients is associated with a significant increase in anxiety and depression severity. Routine psychological assessment and psycho-oncological support should be implemented as integral components of oncological care, with particular attention to patients in disadvantaged socioeconomic conditions.

## 1. Introduction

Breast cancer is the most commonly diagnosed malignant neoplasm among women worldwide and remains the leading cause of cancer-related mortality in the female population [1]. Advances in systemic therapy, particularly chemotherapy, have significantly improved patient survival and prognostic outcomes [2]. However, the cancer diagnosis itself, along with the burdens of intensive cytotoxic treatment, imposes substantial psychological distress. The literature consistently highlights that breast cancer patients experience considerable emotional disturbances, with anxiety and depression being the most frequently reported disorders [3,4]. The scale of this issue is considerable—epidemiological studies estimate that 30% to 50% of patients present with clinically significant mood disorders [5]. These symptoms may manifest shortly after diagnosis, throughout treatment, or even following its completion [6]. In the initial stages of therapy, anxiety often predominates over depression, emerging as a response to the recent diagnosis, the necessity of commencing treatment, and fear of adverse effects. In contrast, depression tends to intensify over subsequent months, correlating with the depletion of psychological resources, the cumulative toxicity of treatment, and limitations in daily functioning [7].

The application of appropriate psychological models enables a better understanding of the emotional processes accompanying cancer and chemotherapy treatment. Lazarus and Folkman’s stress-coping model emphasizes the importance of cognitive appraisal of the illness and individual adaptive strategies in shaping patients’ emotional responses. From a cognitive-behavioral perspective, cognitive distortions—such as catastrophizing or pessimistic interpretation of symptoms—are considered key factors contributing to the persistence of depressive and anxiety symptoms [8]. In turn, Engel’s biopsychosocial model emphasizes the interplay between biological, psychological, and social factors in the processes of illness and recovery. This perspective is particularly relevant in the context of breast cancer—a disease with a profound emotional and social dimension [9].

Chemotherapy, as one of the most invasive forms of systemic treatment, plays a pivotal role in shaping the psychological well-being of breast cancer patients. Side effects such as nausea, vomiting, hair loss, chronic fatigue, and cognitive disturbances can significantly impair quality of life and contribute to the onset of depression [10]. At the same time, chemotherapy offers hope for remission or life extension, which may alleviate anxiety symptoms in some patients [11]. However, clinical practice shows that, for many individuals, repeated hospital visits, exposure to the suffering of other patients, and the cyclical nature of treatment intensify feelings of helplessness and diminish psychological well-being [12].

International studies underscore the importance of routinely monitoring anxiety and depression in women undergoing treatment for breast cancer. Burgess et al. [6] reported that over 40% of patients experience high levels of anxiety during the first year following diagnosis, while depressive symptoms tend to escalate during the second and third years of follow-up. Mitchell et al. [5], in a meta-analysis, demonstrated that emotional disorders are significantly more prevalent in this patient group than in the general population. Similar findings were reported by Mehnert et al. [7], who emphasized the necessity of continuous psychological assessment at various stages of oncological care.

The clinical relevance of assessing anxiety and depression in breast cancer patients undergoing chemotherapy is unequivocal. Emotional disturbances in this population are associated with lower quality of life, reduced adherence to therapeutic regimens, and poorer prognostic outcomes [13]. Consequently, international literature increasingly emphasizes the need for routine psychological screening and the integration of psycho-oncological support within comprehensive cancer care [14].

Given these considerations, studies evaluating anxiety and depression levels in women undergoing breast cancer treatment—particularly in the period immediately before and after neoadjuvant chemotherapy—are not only justified but essential. Such investigations enable the identification of emotional change dynamics, help delineate high-risk patient groups, and inform the implementation of targeted psychological interventions. In the context of the Polish healthcare system, where psycho-oncological support is not yet universally standardized, these studies may serve as a foundation for developing clinical guidelines and shaping health policy.

It should be emphasized that, despite progress in recent years, psycho-oncological care in Poland continues to face numerous challenges related to accessibility, quality, and integration within the healthcare system. According to recent studies, access to professional psycho-oncological services remains uneven, and cancer patients frequently encounter barriers stemming from insufficient human and financial resources [15]. Recent studies indicate that, despite growing awareness of its importance, psycho-oncology is still insufficiently integrated into the standard treatment pathway for cancer patients. The situation is further complicated by the fact that the profession of psycho-oncologist in Poland lacks adequate legal regulation. Current systemic solutions include inconsistent regulations regarding the acquisition of professional qualifications, which leads to organizational confusion and, ultimately, limits access to services for oncology patients and their families provided by specialists in this demanding field [16]. It should also be noted that psychological support for cancer patients is not available in outpatient clinics, as Poland has yet to establish an ambulatory specialist care system in the field of psycho-oncology.

Currently, patients can access psycho-oncological assistance only during hospitalization.

The aim of this study is to assess and compare the severity of anxiety and depression in women before and after neoadjuvant chemotherapy for breast cancer, and to identify potential factors associated with emotional disturbances.

## 2. Materials and Methods

### 2.1. Inclusion Criteria for the Study

A total of 211 women diagnosed with breast cancer were enrolled in the study. All participants were treated at the Podkarpackie Oncology Center named after Fr. B. Markiewicz in Brzozów, Poland. Ethical approval for the study was granted by the Bioethics Committee of the Jan Grodek State University in Sanok (approval no. 3/2022), and administrative consent was obtained from the management of the Podkarpackie Oncology Center. Prior to participation, all patients were informed about the nature and objectives of the study and were assured of the voluntary nature of participation, anonymity, and the right to withdraw at any stage without consequences.

Inclusion criteria were as follows: age ≥ 18 years, clinically confirmed stage I–III breast cancer, initiation of neoadjuvant chemotherapy comprising at least four cycles of taxane- and/or anthracycline-based regimens, and no prior systemic treatment for cancer. Patients in the first stage of the disease were treated with neoadjuvant chemotherapy due to a diagnosis of breast cancer with HER2 overexpression or triple-negative breast cancer, with a tumor size greater than 10 mm. Patients were excluded if they had difficulties understanding the questionnaire or communicating in Polish, had a history of psychiatric illness, were diagnosed with metastatic disease, or did not provide informed consent.

It was assumed that all patients treated for breast cancer at the Podkarpackie Oncology Center during the data collection period who met the inclusion criteria specified in Section 2 and provided informed consent to participate in the study would be enrolled. It was considered that a group of patients from a single center constitutes a representative sample, as standardized diagnostic and therapeutic procedures for breast cancer treatment are applied throughout Poland. Therefore, it can be assumed that the adopted recruitment approach ensures the reliability of the obtained results and allows for their generalization to the population of all breast cancer patients in Poland.

### 2.2. Research Tools and Study Settings

To evaluate levels of anxiety and depression, the Hospital Anxiety and Depression Scale (HADS), developed by Zigmond and Snaith [17], was employed. The scale comprises 14 items, with seven questions assessing symptoms of anxiety (HADS-A) and seven addressing depressive symptoms (HADS-D). Each item is scored on a 4-point Likert scale ranging from 0 to 3, yielding a maximum of 21 points per subscale. The following cut-off scores were used to interpret the results:

- 0–7 points: no symptoms;
- 8–10 points: borderline case;
- 11–21 points: clinically significant symptoms of anxiety or depression.

Participants also completed a brief, custom-designed sociodemographic questionnaire. Data collection occurred at two time points: one week prior to the initiation of chemotherapy, and three weeks following the completion of the final chemotherapy cycle. Clinical data were extracted from medical records. The study was conducted in a clinical setting under the supervision of qualified medical staff.

### 2.3. Statistical Analysis

Statistical analyses were conducted using STATISTICA software, version 13. Descriptive statistics—including arithmetic mean, median, maximum and minimum values, standard deviation, and lower and upper quartiles—were used to summarize the distribution of anxiety and depression scores across the study population. Results are presented separately for assessments conducted before and after chemotherapy, as well as for changes observed between the two time points. In our study, statistical analyses were performed only for complete cases (complete-case analysis).

The Wilcoxon signed-rank test was applied to assess differences between paired measurements due to the non-parametric nature of the data. Statistical significance was considered at *p* < 0.05. The following notation was used: *p* < 0.05 (*), *p* < 0.01 (**), *p* < 0.001 (***).

To enhance interpretation, effect sizes (r) for the Wilcoxon test were estimated as the ratio of the standardized test statistic (Z) to the square root of the sample size (√N), in accordance with current methodological guidelines. Estimated values of r ranged from small to moderate (e.g., ~0.23 for anxiety), indicating meaningful clinical effects.

Missing data were handled using a complete case analysis approach. Participants with incomplete HADS questionnaires at either time point were excluded from the paired comparison.

The study sample was based on the number of eligible patients available during the data collection period; therefore, no a priori power or sample size calculation was performed. Consequently, the analyses were not specifically powered to detect differences between subgroups, which should be considered when interpreting the findings.

## 3. Results

### 3.1. Sociodemographic Characteristics of the Study Group

The study sample consisted of 211 women aged between 32 and 84 years (mean age: 56 years). The majority of participants resided in rural areas and were in a stable relationship. The most commonly reported level of education was secondary or post-secondary (43.1%). In terms of occupational status, the two predominant categories were white-collar employment (31.8%) and unemployment (30.3%). Most patients rated their financial situation as satisfactory or good. The vast majority of women were mothers, most frequently of two children, and in over half of the cases, the age at first pregnancy fell within the 19–25 age range. Clinically, the group was primarily composed of patients diagnosed at an early stage of disease, with stage I breast cancer being the most prevalent (84.4%). Detailed demographic and clinical characteristics are presented in Table 1.

### 3.2. HADS—A Anxiety Scale

**Figure 1:**
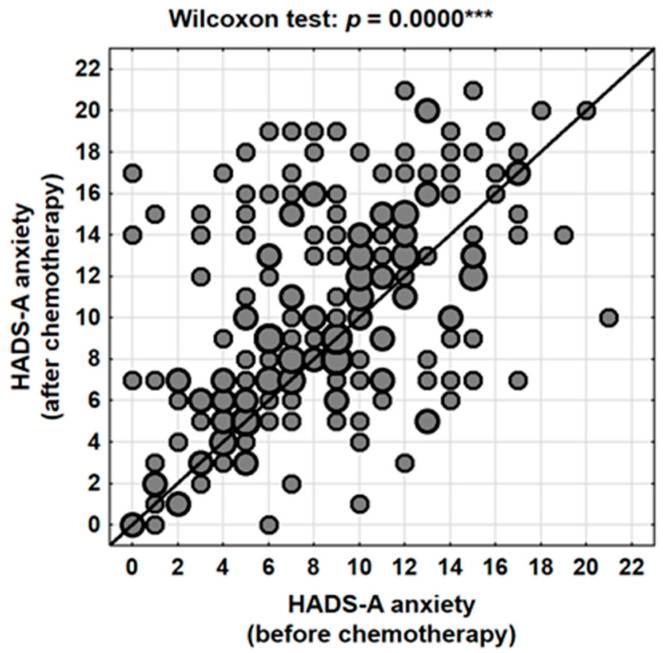
Changes in anxiety levels (HADS-A) in the patients studied depending on the stage of treatment (before vs. after chemotherapy). ***—*p* < 0.001.

Analysis of scores obtained using the HADS-A subscale revealed that chemotherapy had a statistically significant impact on levels of perceived anxiety. The mean anxiety score prior to treatment was 8.6 points (95% CI: 8.0–9.2), which increased to 10.3 points (95% CI: 9.6–11.0) following the completion of chemotherapy. This difference was statistically significant (Wilcoxon signed-rank test, *p* < 0.001), with a mean increase of 1.7 points (95% CI: 1.1–2.4) (Table 2, Figure 1).

It is worth noting that although the absolute values did not reach the upper limits of the scale, even a moderate increase may carry substantial clinical relevance—particularly in a population of patients already burdened both physically and mentally by the demands of oncological treatment.

Individual-level analysis indicated that anxiety severity increased in 58.3% (N = 123) of women following chemotherapy, decreased in 27.0% (N = 57), and remained unchanged in 14.7% (N = 31) of cases. These findings suggest that while the majority of patients experienced a worsening of anxiety symptoms, a subset of women reported improvement—possibly reflecting a sense of hope and reassurance associated with the initiation and completion of active treatment.

Clinical categorization of HADS-A scores further confirmed a worsening trend in anxiety levels. The proportion of women scoring within the normal range decreased from 43.1% before chemotherapy to 34.1% after treatment, while the percentage of patients falling within the pathological range increased from 32.7% to 46.4% (Table 3). Consequently, nearly one in two patients post-chemotherapy qualified for psychological intervention based on their anxiety scores.

### 3.3. HADS—D Measure of Depression

**Figure 2:**
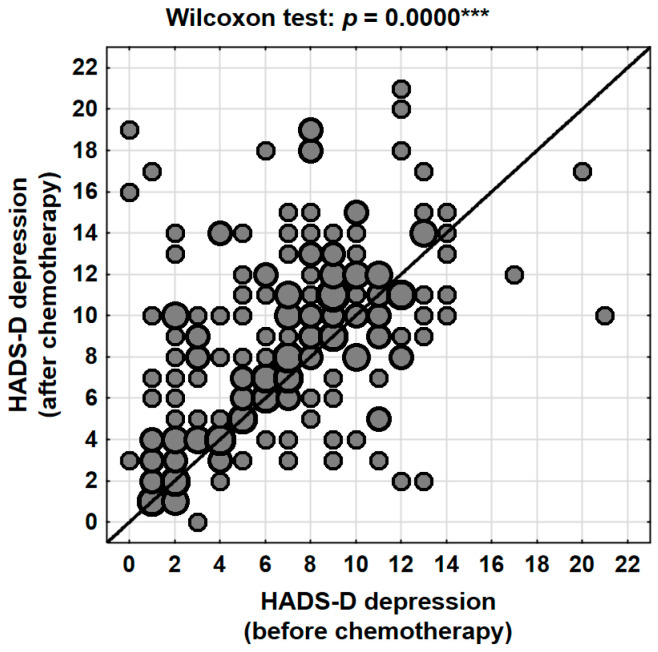
Changes in depression levels (HADS-D) in the patients studied depending on the stage of treatment (before vs. after chemotherapy). ***—*p* < 0.001.

The mean depression score prior to chemotherapy, as measured by the HADS-D subscale, was 7.0 points (95% CI: 6.5–7.5), with a median of 7 and a standard deviation of 4.0 (range: 0–21). Following the completion of treatment, the mean score increased to 8.8 points (95% CI: 8.2–9.4), with a median of 9 and a standard deviation of 4.4 (range: 0–21). This change was highly statistically significant (*p* < 0.001, Wilcoxon signed-rank test), with a mean increase of 1.8 points (95% CI: 1.2–2.4) (Table 4, Figure 2).

At the individual level, 59.7% (N = 126) of patients experienced an increase in depressive symptoms following the completion of chemotherapy. In contrast, 23.2% (N = 49) of participants showed a decrease in their HADS-D scores, while 17.1% (N = 36) reported no change. These findings indicate that nearly six out of ten women experienced a deterioration in their psychological well-being in terms of depression, highlighting the significant emotional impact of systemic therapy.

The clinical classification of HADS-D scores further corroborated these findings. The proportion of patients with scores within the normal range (≤7 points) decreased from 54.5% before chemotherapy to 38.9% after treatment. At the same time, the percentage of patients with clinically abnormal levels of depression (≥11 points) nearly doubled, rising from 19.4% prior to treatment to 35.5% following chemotherapy. The proportion of participants with borderline results (8–10 points) remained relatively stable (26.1% vs. 25.6%) (Table 5). This pronounced shift in score distribution indicates that chemotherapy acts as a factor exacerbating depressive symptoms and, in many cases, contributes to the transition of patients from borderline to clinically significant levels of depression.

### 3.4. Factors Influencing the Severity of Anxiety and Depression

No statistically significant differences were observed in anxiety or depression levels between participants residing in rural and urban areas, either before or after chemotherapy. This finding indicates that place of residence was not a determining factor influencing emotional status within the studied population (Table 6A).

Patients in a relationship demonstrated lower mean post-treatment depression scores compared with single women (8.5 vs. 9.9 points). However, this difference did not reach statistical significance, suggesting only a potential trend toward a protective effect of partner support (Table 6B).

Financial situation emerged as a significant differentiating factor. Women who reported poor material conditions had higher post-chemotherapy HADS-A (13.5 vs. 9.9; *p* = 0.0004) and HADS-D (11.6 vs. 8.4; *p* = 0.0013) scores compared with those who rated their financial situation as good. These differences were also present before treatment, although they did not reach statistical significance at that time. These findings indicate that low socioeconomic status may substantially increase vulnerability to the development of emotional disturbances during oncological therapy. However, it should be noted that the assessment of financial condition was subjective, which constitutes a limitation in interpreting these results (Table 6C).

Comparison between patients with stage I breast cancer and those with stage II–III disease revealed no statistically significant differences in anxiety or depression levels, either before or after chemotherapy. The absence of association may reflect both the relatively small size of the group with more advanced disease (stage III) and the fact that subjective experiences of anxiety and depression are not necessarily proportional to the clinical stage of cancer (Table 6D).

## 4. Discussion

The results of the present study clearly demonstrate that chemotherapy in women with breast cancer is associated with an increase in the severity of both anxiety and depressive symptoms. In both dimensions, mean HADS scores were significantly higher after treatment than before its initiation, and the proportion of patients with clinically abnormal results increased substantially. These findings are consistent with previous international reports indicating a high prevalence of emotional disturbances among oncology patients [5,6,7].

In a meta-analysis conducted by Mitchell et al. [5], symptoms of depression and anxiety were found in approximately 30–40% of cancer patients, with the highest intensity observed among those with breast cancer. Similarly, Krebber et al. [18] reported that the prevalence of depression among oncology patients reached 24.6% when assessed using structured diagnostic interviews and 31.6% when using self-report instruments, highlighting both the high frequency of affective disorders and the variability related to the assessment method. Consistent findings were also reported by Tsaras et al. [19], who observed clinically significant symptoms of anxiety in 47% and depression in 39% of women with breast cancer, and by Gopalan et al. [20], who found that approximately 41% of cancer patients met the criteria for a psychiatric disorder, most commonly depressive and anxiety disorders. Likewise, Burgess et al. [6], in a five-year longitudinal study, reported high levels of anxiety during the first year following diagnosis, accompanied by a progressive increase in depressive symptoms over subsequent years. Our findings align with this trend, suggesting that intensive systemic treatment represents a particularly burdensome stage of therapy, during which the risk of emotional disturbances is greatest.

Several studies with comparable methodology have evaluated anxiety and depression using the HADS at two time points—before and after chemotherapy. Linden et al. [21] observed a significant increase in depressive symptoms after several treatment cycles, while anxiety levels gradually intensified from the onset of therapy. Lemieux et al. [22] emphasized that anxiety predominates prior to chemotherapy initiation, whereas depression more commonly develops in later treatment phases. Similarly, Watson et al. [23] found that nearly half of their participants reported higher HADS-D scores following chemotherapy, a finding reflected in our observation of a marked rise in the proportion of women falling within the clinically abnormal category. Leung et al. [24] also noted increases in both HADS-A and HADS-D scores after chemotherapy among breast cancer patients. Although some improvement may occur in certain individuals as a result of psychological adaptation, the overall trend at the group level points toward a deterioration in emotional well-being.

Our findings also underscore the relevance of socioeconomic factors. Women who reported poor financial conditions demonstrated significantly higher HADS scores for both anxiety and depression after chemotherapy. These results are consistent with those of Carreira et al. [4], who highlighted that low socioeconomic status significantly increases the risk of developing psychological disorders in women with breast cancer. Similar trends were noted by Bargon et al. and Chou et al., emphasizing that both pandemic-related stressors [25,26] and the lack of social support in younger women can exacerbate psychological distress. Kroenke et al. [13] further noted that lack of social support and limited material resources may negatively affect treatment adherence and quality of life. Our study confirms these associations within a Polish patient population.

From a biopsychosocial perspective, it is important to emphasize that, in the Polish cultural context, the experience of cancer is often accompanied by a strong sense of stigmatization and limited access to professional psychological support within the public healthcare system. Studies conducted by de Walden-Gałuszko [27]. Studies conducted by de Walden-Gałuszko indicate that, although awareness of the need for psycho-oncological care is increasing, many oncology centers in Poland still lack an adequate number of specialists and structured programs for emotional support. Additionally, cultural factors—such as a tendency to avoid discussions about emotions, the belief that one must “stay strong” in the face of illness, and traditional gender roles—may limit patients’ willingness to seek psychological assistance. Institutional support in Poland, while gradually developing (e.g., through the efforts of the Polish Psycho-Oncology Society), remains fragmented and often dependent on local initiatives. As a result, these factors may intensify stress and helplessness, thereby increasing the risk of emotional disturbances during chemotherapy [27,28].

Importantly, place of residence, marital status, and disease stage did not significantly differentiate anxiety or depression levels. This suggests that the psychological consequences of chemotherapy are a universal experience, largely independent of basic demographic or clinical characteristics. Similar conclusions were drawn by Mehnert et al. [7], who reported that the prevalence of emotional disturbances among cancer patients is not always correlated with disease stage, but rather with subjective perceptions of threat and limited adaptive resources.

The exacerbation of anxiety and depressive symptoms is not merely a psychological issue. As noted by Lawrence et al. and Walker et al. [10,12], emotional disturbances can lead to poorer treatment tolerance, decreased adherence to therapy, and even greater susceptibility to chemotherapy-related adverse effects due to dysregulation of the hypothalamic–pituitary–adrenal (HPA) axis and immune dysfunction. These findings are further corroborated by Bower [29] and Naser et al. [30], who emphasized that persistent emotional distress in cancer patients not only contributes to fatigue and impaired immune function but also significantly deteriorates overall treatment outcomes. Consequently, the absence of adequate psychological intervention may worsen both quality of life and clinical outcomes.

From a practical perspective, the findings emphasize the necessity of routine psychological assessment at multiple stages of oncological care. According to the National Comprehensive Cancer Network (NCCN) guidelines [14], screening for depression and anxiety should be integrated into standard cancer care to ensure early identification and intervention for those in need of support. This is particularly crucial for patients with limited financial resources, who—based on our findings—are at the highest risk for developing severe emotional disturbances.

### Study Limitations

Several limitations of this study should be acknowledged. First, the research relied exclusively on a self-report method (HADS), which, although validated and widely used in international studies [17], remains susceptible to subjective bias. Second, the study included only two measurement points (before and after chemotherapy), which limits the ability to fully capture the long-term dynamics of emotional changes. Third, the assessment of financial situation was subjective—patients experiencing higher levels of emotional distress may have evaluated their material resources more pessimistically. Additionally, potential selection bias cannot be excluded, as all participants were recruited from a single oncology center, which may limit the generalizability of the findings. The absence of control for relevant clinical variables, such as type of chemotherapy regimen or coexisting somatic conditions, also constitutes a limitation that may have influenced emotional outcomes. Nonetheless, the study provides contextually relevant insights into psycho-oncological needs within the Polish healthcare system, highlighting practical implications for patient support rather than claiming conceptual novelty.

Moreover, in this study, the impact of variables such as comorbidities, prior psychiatric history (beyond the applied exclusion criteria), and the type and duration of chemotherapy on anxiety and depression levels was not analyzed. These factors could potentially influence the levels of anxiety and depression in the studied group and should be considered when interpreting the results. In future research, it would be advisable to include these variables in the statistical analysis or to use a more homogeneous study group in order to minimize the impact of these confounding factors. It should also be noted that the assessment of mental status was conducted three weeks after the completion of chemotherapy, that is, during a period of possible increased physical toxicity. Symptoms such as fatigue or weakness may overlap with depressive symptoms assessed using the HADS. Therefore, the increase in HADS scores may partially result from transient somatic symptoms rather than solely from changes in the patients’ psychological condition.

## 5. Conclusions

Despite certain limitations, our findings are consistent with international research on the psychological vulnerability of women undergoing breast cancer treatment and support the validity of the Hospital Anxiety and Depression Scale (HADS) as a reliable screening tool for emotional disturbances during chemotherapy. The significant increase in anxiety and depressive symptoms post-treatment reflects not only statistical relevance but also meaningful clinical impact, particularly for patients with socioeconomic disadvantage—the strongest predictor of psychological distress in our cohort. These outcomes underscore the necessity of integrating routine psychological assessment and tailored psycho-oncological care into standard oncology protocols. Prioritizing emotional support, especially for vulnerable populations, may improve treatment adherence, enhance quality of life, and ultimately influence clinical outcomes. Future longitudinal studies should further explore the emotional trajectory across all stages of therapy to refine personalized care models.

In this context, the implementation of standardized psychological screening protocols should become a routine component of cancer care. Multidisciplinary coordination—including oncologists, psycho-oncologists, and support staff—is essential to ensure timely identification of emotional needs and effective therapeutic response. This recommendation is substantiated by the statistically significant pre- and post-treatment differences observed in our study.

## Figures and Tables

**Table 1:** Characteristics of the study group.

Characteristics				
	Mean	Median (Quartiles)	SD	Range
Age [years]	56.22	56 (48–65)	11.03	32–84
Weight [kg]	70.87	70 (62–80)	13.02	45–110
Height [cm]	161.46	162 (158–165)	6.44	143–180
BMI [kg/m^2^]	27.15	26.6 (23.7–29.9)	4.62	18.5–41.7
Characteristics		N	%	
BMI				
Normal weight		77	36.49	
Overweight		82	38.86	
Obesity		52	24.64	
Place of residence				
Rural area		117	55.45	
City up to 10 th. inhab.		36	17.06	
City 10–100 th. inhab.		51	24.17	
City over 100 th. Inhab.		7	3.32	
Marital status				
Single		36	17.06	
In relationship		175	82.94	
Education level				
Primary/secondary		11	5.21	
Vocational		44	20.85	
Secondary/post-secondary		91	43.13	
Bachelor’s/master’s degree		65	30.81	
Financial standing				
Very good		11	5.21	
Good		174	82.46	
Bad		26	12.32	
Professional activity				
Physical work		37	17.54	
Office work		67	31.75	
Retirement/invalidity pension		39	18.48	
Unemployed		64	30.33	
Other sources of income		4	1.90	
Children				
No children		26	12.32	
1 child		28	13.27	
2 children		91	43.13	
3 children		50	23.70	
4 or more children		16	7.58	
Pregnancies				
No pregnancies		20	9.48	
1 pregnancy		29	13.74	
2 pregnancies		80	37.91	
3 pregnancies		51	24.17	
4 pregnancies		22	10.43	
5 or more pregnancies		9	4.27	
Age at first pregnancy				
No pregnancies		20	9.48	
Up to 18 years		24	11.37	
19–25 years		122	57.82	
26–30 years		35	16.59	
31–35 years		10	4.74	
Cancer stage				
Stage I		31	14.69	
Stage II		178	84.36	
Stage III		2	0.95	
Total		211	100	

**Table 2:** Comparison of anxiety severity (HADS-A) in patients before and after chemotherapy.

Anxiety (HADS)	Mean (with 95% CI)	Median	Std. Dev.	Min	Max
before chemotherapy	8.6 (8.0; 9.2)	9	4.5	0	21
after chemotherapy	10.3 (9.6; 11.0)	10	5.1	0	21
change (*p* = 0.0000 ***)	1.7 (1.1; 2.4)	1	4.8	−11	17

***—*p* < 0.001.

**Table 3:** Percentage of patients with normal, borderline, and abnormal anxiety levels (HADS-A) before and after treatment.

Anxiety Level	Before Chemotherapy		After Chemotherapy	
	N	%	N	%
normal	91	43.1%	72	34.1%
borderline	51	24.2%	41	19.4%
abnormal	69	32.7%	98	46.4%

**Table 4:** Comparison of depression severity (HADS-D) in patients before and after chemotherapy.

Depression (HADS—D)	Mean (with 95% CI)	Median	Std. Dev.	Min	Max
Before chemotherapy	7.0 (6.5; 7.5)	7	4.0	0	21
After chemotherapy	8.8 (8.2; 9.4)	9	4.4	0	21
change (*p* = 0.0000 ***)	1.8 (1.2; 2.4)	1	4.4	−11	19

***—*p* < 0.001.

**Table 5:** Percentage of patients with normal, borderline, and abnormal levels of depression (HADS-D) before and after treatment.

Level of Depression	Before Chemotherapy		After Chemotherapy	
	*N*	%	*N*	%
normal	115	54.5%	82	38.9%
borderline	55	26.1%	54	25.6%
abnormal	41	19.4%	75	35.5%

**Table 6:** Measures of anxiety and depression before and after chemotherapy, and place of residence (**A**), marital status (**B**), financial situation (**C**), and cancer stage (**D**).

**A**	**HADS Measures**		**Place of Residence (A)**				***p***
			**Rural Area (N = 117)**		**Urban Area (N = 94)**		
			**Mean (with 95% CI)**	**Median**	**Mean (with 95% CI)**	**Median**	
	Before chemotherapy	anxiety	8.5 (7.6; 9.3)	8	8.8 (7.9; 9.7)	9	ns
		depression	7.0 (6.2; 7.8)	7	7.0 (6.3; 7.7)	7	ns
	After chemotherapy	anxiety	10.0 (9.0; 10.9)	9	10.8 (9.8; 11.8)	11	ns
		depression	8.6 (7.8; 9.4)	9	9.0 (8.1; 10.0)	9	ns
	Change after chemotherapy	anxiety	1.5 (0.7; 2.3)	1	2.0 (0.9; 3.1)	2	ns
		depression	1.6 (0.8; 2.4)	1	2.0 (1.1; 2.9)	1	ns
**B**	**HADS Measures**		**Marital Status (B)**				***p***
			**Single (N = 36)**		**in a Relationship (N = 175)**		
			**Mean (with 95% CI)**	**Median**	**Mean (with 95% CI)**	**Median**	
	Before chemotherapy	anxiety	9.5 (7.7; 11.4)	9.5	8.4 (7.8; 9.1)	9	ns
		depression	7.9 (6.3; 9.6)	7.5	6.8 (6.2; 7.4)	7	ns
	After chemotherapy	anxiety	11.1 (9.4; 12.9)	11.5	10.2 (9.4; 10.9)	9	ns
		depression	9.9 (8.2; 11.5)	10	8.5 (7.9; 9.2)	9	ns
	Change after chemotherapy	anxiety	1.6 (−0.1; 3.3)	0	1.7 (1.0; 2.4)	1	ns
		depression	1.9 (0.1; 3.7)	1	1.8 (1.1; 2.4)	1	ns
**C**	**HADS Measures**		**Financial Situation (C)**				***p***
			**Good (N = 185)**		**Bad (N = 26)**		
			**Mean (with 95% CI)**	**Median**	**Mean (with 95% CI)**	**Median**	
	Before chemotherapy	anxiety	8.4 (7.7; 9.0)	8	10.4 (8.3; 12.5)	10	ns
		depression	6.8 (6.2; 7.3)	7	8.5 (6.3; 10.8)	8	ns
	After chemotherapy	anxiety	9.9 (9.2; 10.6)	9	13.5 (11.8; 15.3)	14	0.0004 ***
		depression	8.4 (7.8; 9.0)	8	11.6 (9.7; 13.5)	11	0.0013 **
	Change after chemotherapy	anxiety	1.5 (0.9; 2.2)	1	3.1 (0.5; 5.7)	3	ns
		depression	1.6 (1.0; 2.2)	1	3.1 (0.3; 5.9)	2.5	ns
**D**	**HADS Measures**		**Cancer Stage (D)**				***p***
			**I (N = 31)**		**II–III (N = 180)**		
			**Mean (with 95% CI)**	**Median**	**Mean (with 95% CI)**	**Median**	
	Before chemotherapy	anxiety	8.7 (8.0; 9.3)	9	8.4 (6.8; 10.1)	8	ns
		depression	7.0 (6.4; 7.6)	7	6.8 (5.3; 8.3)	6	ns
	After chemotherapy	anxiety	10.2 (9.5; 11.0)	10	10.9 (8.8; 12.9)	11	ns
		depression	8.6 (8.0; 9.3)	9	9.6 (7.8; 11.3)	10	ns
	Change after chemotherapy	anxiety	1.6 (0.9; 2.3)	1	2.5 (0.7; 4.2)	1	ns
		depression	1.6 (1.0; 2.2)	1	2.8 (1.0; 4.5)	2	ns

ns—not significant (*p* > 0.05), **—*p* < 0.01, ***—*p* < 0.001.

## Data Availability

Data can be obtained by contacting the corresponding author.

## Abbreviations

The following abbreviations are used in this manuscript:

HADS	the Hospital Anxiety and Depression Scale
NCCN	National Comprehensive Cancer Network
